# Postdischarge Intervention for Stroke Caregivers: Protocol for a Randomized Controlled Trial

**DOI:** 10.2196/21799

**Published:** 2020-11-11

**Authors:** Jennifer H LeLaurin, Avi H Lamba, Nathaniel D Eliazar-Macke, Magda K Schmitzberger, I Magaly Freytes, Stuti Dang, W Bruce Vogel, Charles E Levy, S Angelina Klanchar, Rebecca J Beyth, Ronald I Shorr, Constance R Uphold

**Affiliations:** 1 North Florida/South Georgia Veterans Health System Gainesville, FL United States; 2 Department of Health Outcomes and Biomedical Informatics College of Medicine University of Florida Gainesville, FL United States; 3 Flint Hill School Oakton, VA United States; 4 Geriatric Research Education and Clinical Center North Florida/South Georgia Veterans Health System Gainesville, FL United States; 5 Geriatric Research Education and Clinical Center Miami VA Healthcare System Miami, FL United States; 6 Miller School of Medicine University of Miami Miami, FL United States; 7 Physical Medicine and Rehabilitation Service North Florida/South Georgia Veterans Health System Gainesville, FL United States; 8 Department of Occupational Therapy and Center for Arts in Medicine University of Florida Gainesville, FL United States; 9 James A Haley Veterans Hospital Tampa, FL United States; 10 Division of General Internal Medicine Department of Medicine University of Florida Gainesville, FL United States; 11 Department of Aging and Geriatric Research College of Medicine University of Florida Gainesville, FL United States

**Keywords:** COVID-19, stroke, caregivers, depression, burden, randomized controlled trial, web-based intervention, problem-solving

## Abstract

**Background:**

The majority of stroke survivors return to their homes and need assistance from family caregivers to perform activities of daily living. These increased demands coupled with the lack of preparedness for their new roles lead to a high risk for caregivers developing depressive symptoms and other negative outcomes. Follow-up home support and problem-solving interventions with caregivers are crucial for maintaining stroke survivors in their homes. Problem-solving interventions are effective but are underused in practice because they require large amounts of staff time to implement and are difficult for caregivers logistically.

**Objective:**

The aim of this study is to test a problem-solving intervention for stroke caregivers that can be delivered over the telephone during the patient’s transitional care period (time when the stroke survivor is discharged to home) followed by 8 asynchronous online sessions.

**Methods:**

The design is a two-arm parallel randomized clinical trial with repeated measures. We will enroll 240 caregivers from eight Veterans Affairs (VA) medical centers. Participants randomized into the intervention arm receive a modified problem-solving intervention that uses telephone and web-based support and training with interactive modules, fact sheets, and tools on the previously developed and nationally available Resources and Education for Stroke Caregivers’ Understanding and Empowerment Caregiver website. In the usual care group, no changes are made in the information, discharge planning, or care the patients who have had a stroke normally receive, and caregivers have access to existing VA resources (eg, caregiver support line, self-help materials). The primary outcome is a change in caregiver depressive symptoms at 11 and 19 weeks after baseline data collection. Secondary outcomes include changes in stroke caregivers’ burden, knowledge, positive aspects of caregiving, self-efficacy, perceived stress, health-related quality of life, and satisfaction with care and changes in stroke survivors’ functional abilities and health care use. The team will also determine the budgetary impact, facilitators, barriers, and best practices for implementing the intervention. Throughout all phases of the study, we will collaborate with members of an advisory panel.

**Results:**

Study enrollment began in June 2015 and is ongoing. The first results are expected to be submitted for publication in 2021.

**Conclusions:**

This is the first known study to test a transitional care and messaging center intervention combined with technology to decrease caregiver depressive symptoms and to improve the recovery of stroke survivors. If successful, findings will support an evidence-based model that can be transported into clinical practice to improve the quality of caregiving post stroke.

**Trial Registration:**

ClinicalTrials.gov NCT01600131; https://www.clinicaltrials.gov/ct2/show/NCT01600131

**International Registered Report Identifier (IRRID):**

DERR1-10.2196/21799

## Introduction

Stroke is a leading cause of serious, long-term disability [1]. Most stroke survivors return to their homes and need family members to assist with daily activities such as bathing and toileting [2]. Evidence is accumulating that caregivers play a critically important role in helping survivors recover post stroke [3]. Researchers have found an association of family support with the improvements in the stroke survivors’ physical, psychosocial, and overall functioning [4-6].

Because of the demands of caregiving, family members of stroke survivors are at high risk for developing depressive symptoms, burden, stress, and poor quality of life [7-9]. These negative caregiver outcomes are a major contributor to survivors’ hospital readmission and institutionalization [10-14]. Strokes, unlike most other chronic diseases, occur without warning, and family caregivers need to quickly learn how to care for stroke survivors who have multiple impairments (eg, motor, speech, cognitive, behavioral) while simultaneously adjusting to changes in their own lives [11]. As a result, caregivers usually have feelings of inadequacy in their new roles and many unmet needs [15-18]. Thus, transitional care, follow-up support, and education with caregivers are crucial for maintaining stroke survivors in their homes.

Researchers have consistently found that interventions to help caregivers resolve problems are the most effective in supporting caregivers at home [19,20]. Unfortunately, these problem-solving interventions have been underused in practice because they require large amounts of staff time to implement and are difficult for caregivers who must travel for the intervention or be available for phone calls or visits in the home. To overcome these barriers, stroke caregiver programs are needed that involve low-cost, feasible interventions that are sustainable in routine clinical practice.

Individualized, tailored problem-solving and support programs are more likely to change health behaviors and improve self-efficacy than generic programs [19,21]. Bakas et al [22] conducted a randomized controlled trial (RCT) of an 8-week program using a telephone support approach and a task skill-building kit (TASK II), and found that the intervention improved depressive symptoms and other outcomes among caregivers with mild to severe depressive symptoms. King et al [23] conducted an RCT of a 10-session problem-solving intervention delivered in-person and by telephone. This study found improvements in caregiver depression, perception of life changes, and health at 3 months, though the improvements were not sustained at 6 months [23]. Although these interventions were effective in improving stroke caregiver outcomes, they were conducted in-person or by telephone and were, therefore, labor intensive and required scheduling to meet the convenience needs of the caregivers. For these reasons, these problem-solving interventions have been underused in practice.

Telehealth technologies offer promising approaches for overcoming traditional barriers to stroke caregiver interventions and improving outcomes. These approaches allow clinicians and researchers to provide health services through technologies such as the internet and online messaging, either alone or as supplements to enhance in-person or telephone caregiver support and training. The advantage of using internet-based interventions is that adults can receive up-to-date information in a place and time that is convenient for them. To our knowledge, only a few investigators have conducted technology-based interventions for caregivers [24].

One potentially effective technology-based delivery method that has not been well studied is online messaging between providers and patients and their caregivers. Previous researchers found that online messaging enhanced access to care [25], improved quality of care [26], and reduced the cost and use of care [27,28]. Other benefits include patients’ comfort while asking questions and the ability to save messages [29]. Additionally, previous studies found that online messaging is acceptable to patients and improved a variety of patient outcomes [30-32].

To our knowledge, no previous studies have been conducted with online messaging for stroke caregivers. To address gaps in previous research, this study tests a tailored, problem-solving intervention for stroke caregivers that is delivered in one telephone session during the transitional care period (eg, time in which the stroke survivor is discharged home) followed by online messaging center sessions over a secure messaging system. The long-term goal is to develop a model for future caregivers that can be sustainable in routine clinical practice and is not overly burdensome for caregivers.

This study has five aims. The primary aim (aim 1) is to test the effect of the intervention on stroke caregivers’ depressive symptoms at 11 and 19 weeks after baseline data collection. Aim 2 is to test the effect of the intervention on stroke caregivers’ burden, knowledge, positive aspects of caregiving, self-efficacy, perceived stress, health-related quality of life, and satisfaction with care at the posttest assessments. Aim 3 is to test the effect of the intervention on stroke survivors’ outcomes: functional abilities and health care use (ie, unintended hospital stays, emergency room visits). Aim 4 is to determine the budgetary impact of implementing the intervention. Aim 5 is to determine the facilitators, barriers, and best practices for implementing the intervention. Our primary hypothesis is that stroke caregivers who receive the intervention will have fewer depressive symptoms compared to those in the usual care group. Our secondary hypotheses are that caregivers and stroke survivors in the intervention arm will have superior outcomes compared to the usual care arm.

## Methods

### Ethics Approvals and Monitoring

Approvals were obtained from the Veterans Affairs (VA) Central Institutional Review Board and the local VA Rehabilitation and Development committees at the three primary sites (Gainesville, Tampa, and Miami). The protocol is registered in ClinicalTrials.gov (NCT01600131). Informed consent is obtained from all caregivers participating in the study. Although stroke survivors do not directly participate in the study, we discuss their medical history with their caregiver. Therefore, we also obtain informed consent from the stroke survivors. The study is monitored through annual reports to the VA Health Services Research & Development Data Safety Monitoring Board [33]. No interim data analysis has or will be conducted.

### Advisory Panel

We established an advisory panel consisting of clinicians and VA leadership at the national and local level. Initially, monthly conference calls were held to obtain members’ input in the planning and development phases. Later, periodic meetings were held to obtain advice on conducting the study. After the data are collected, panel members will meet to help in the interpretation of the study findings and planning for the dissemination of results and strategies to sustain the intervention in practice if it is found to be successful.

### Study Setting

The original protocol was to conduct the study at three Florida VA medical centers (Gainesville, Tampa, and Miami). These three sites will be referred to as the main study sites. Due to lower than expected initial enrollment, we modified the protocol after initiating the trial to expand recruitment to five additional or remote VA sites: Houston, TX; Richmond, VA; Little Rock, AR; Nashville, TN; and Boston, MA. At the three main sites, staff assist with the management of the study, conduct the intervention, and recruit caregivers from all eight study sites.

### Study Design and Eligibility Criteria

**Figure 1:**
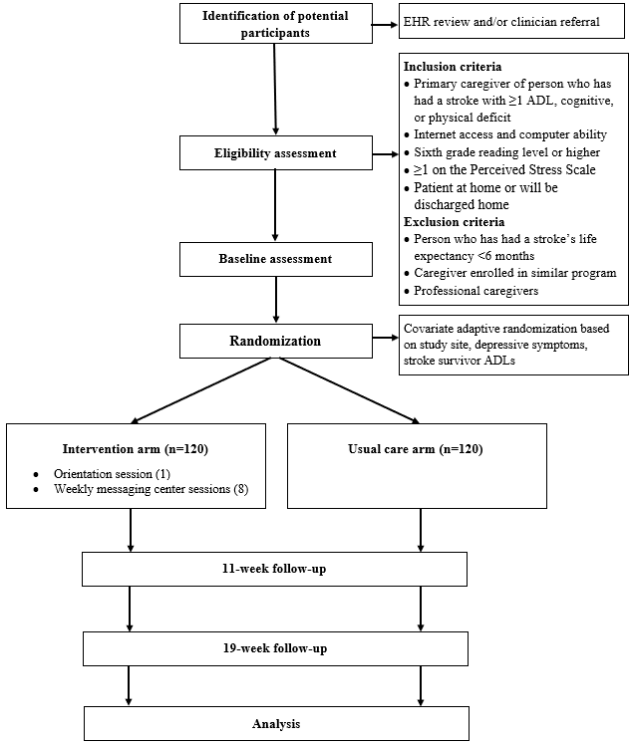
Study flowchart. ADL: activity of daily living; EHR: electronic health record.

This study is a two-arm parallel randomized clinical trial with repeated measures. The study flowchart is presented in Figure 1. All caregivers of stroke survivors are eligible for participation if they meet the following criteria: are the primary caregiver and provide the majority of care for an individual who has a primary diagnosis of stroke (International Statistical Classification of Diseases and Related Health Problems, 10th revision [ICD10] codes for stroke: 160.0-169.998), stroke survivor has ≥1 activity of daily living (ADL) deficit (≤95 on the Barthel Index [34]) or a new or worsening cognitive or physical functioning problem, caregiver has internet and email access and ability, caregiver reads English at the sixth-grade reading level or better (≥13 on the Behavior Rating Inventory of Executive Function Health Literacy Scale [35]), caregiver scores ≥1 on the Perceived Stress Scale [36], and stroke survivor was discharged home within the preceding 4 months or plans to be ultimately discharged home. Eligibility is determined by caregiver self-report and review of the stroke survivor’s electronic health record (EHR).

Caregivers are excluded if the stroke survivors they care for are terminally ill, have a life expectancy of 6 months or less, are prisoners, or are currently enrolled in or have completed similar caregiver interventions. Caregivers are also excluded if they are professional caregivers who have no pre-existing relationships with the stroke survivor. Life expectancy and service use are determined by reviewing the EHR and conferring with the in-patient staff and the investigators’ clinical team members. If an enrolled caregiver no longer meets eligibility criteria (eg, no longer caring for the stroke survivor), they are withdrawn from the study. If an enrolled caregiver is unable to complete the intervention, we still attempt to collect follow-up data from them.

### Recruitment and Enrollment

Recruitment is conducted through two strategies: clinician referral followed by face-to-face recruitment or EHR review followed by telephone recruitment. At the main study sites, clinicians caring for stroke survivors inform study staff of interested participants. Study staff then contact the caregiver either in-person or by phone, explain the study, and screen the stroke survivor and caregiver for eligibility. A second strategy is to identify stroke survivors at all study sites who have a diagnosis of stroke based on ICD10 codes by reviewing the VA Patient Care Encounter Package Dataset and the VA’s EHR. The team mails letters of invitation and self-addressed, stamped recruitment postcards with an opt-in or opt-out option to the stroke survivor or next of kin. A staff member telephones the stroke survivors or their next of kin (ie, those who returned the postcards with positive responses or those who did not return the postcards) and explains the study and determines eligibility.

Staff conduct the informed consent process with eligible participants in person or by telephone.

For patients who have had a stroke who have potentially diminished decision-making capacity, a study staff member asks the patient screening questions to assess their orientation (ie, “what is the month?” “what is the year?” “what state are we in?”) and comprehension (ie, asking the patient to reiterate back their understanding of the study). If patients are unable to answer the screening questions or the clinical staff indicates the potential for diminished decision-making capacity, we obtain consent from the patient’s legally authorized representative. We obtain assent of the patients who have had a stroke and respect any dissent of the patients. If patients have physical disabilities and are unable to sign the informed consent forms, the patients are asked to make marks with witnesses present. If patients who have had a stroke are aphasic or unable to speak clearly, we confer with the patient’s clinical staff regarding their decision-making capacity.

Study staff at the main study sites are responsible for all recruitment and enrollment activities. Only caregivers participate in data collection and the intervention. Caregivers receive a US $20 incentive for each data collection session they participate in (US $60 total). Caregivers selected for qualitative interviews receive an additional US $20 incentive.

### Randomization

After the baseline assessment, caregivers are randomly assigned to one of the two study groups (ie, intervention or usual care) using the Pocock-Simon covariate adaptive randomization procedure [37] that is overseen by the study statistician. This randomization procedure is used to allocate an approximately equal number of caregivers assigned to the two groups within each covariate level (ie, depressed or not, high or low ADL, and study site). The technique has demonstrated an ability to provide good marginal balance in covariates and is frequently used in clinical trials [38].

### Intervention Arm

We are conducting a nurse-guided intervention. In the original study proposal, the first intervention session was planned to occur face-to-face in the in-patient setting shortly before the stroke survivor’s discharge from the hospital. However, because many stroke survivors have a short inpatient stay or are discharged to rehabilitation facilities across a large geographic region, we modified the protocol to begin the intervention after the stroke survivor’s discharge home. Thus, the original intervention manual was modified to include two parts: a telephone orientation component and online messaging center component. These changes were made prior to initiation of recruitment.

The intervention is theoretically based on D’Zurilla and Nezu’s [39] relational and problem-solving model of stress, and incorporates constructs from Lazarus and Folkman’s [40] stress appraisal and coping theory. The model was subsequently refined by Houts and colleagues [41,42] and summarized by Creativity, Optimism, Planning, and Expert Information (COPE). This model emphasizes creative thinking to view problems in new ways, maintaining an optimistic attitude, developing a plan to solve problems, and learning how to seek expert information. Throughout the intervention, the nurses use motivational interviewing techniques [43].

#### Resources and Education for Stroke Caregivers’ Understanding and Empowerment Website

The Resources and Education for Stroke Caregivers’ Understanding and Empowerment (RESCUE) website [44] serves as the foundation for the entire intervention. Consistent with the COPE model theory, the website uses the theme of RESCUE to illustrate how caregivers act as “lifeguards” and are responsible for the safety and well-being of those under their watchful care. Following this theme, the image of a life preserver is used as a branding image and integrated throughout the website. The website has been extensively pretested and evaluated [45]. The website is written in both English and Spanish languages, and includes the following sections: list of resources, a library of patient education newsletters, self-help tools, a glossary of medical terms with phonetic pronunciations, testimonials from stroke caregivers, links to other stroke and caregiver websites, more than 45 fact sheets, a problem-solving training module, and a problem-solving diary form.

A major part of the intervention is teaching from the comprehensive library of over 45 fact sheets. The fact sheets are organized by the COPE framework and include the following: basic information about the problem (eg, common signs and symptoms that caregivers should look for), information that the problem is common but that there are effective treatments, helpful tips for dealing with the problem, and information on when caregivers should seek emergency help or (if less severe symptoms exist) when to call their health care providers. A list of RESCUE website fact sheets is presented in Textbox 1.

**Resources and Education for Stroke Caregivers’ Understanding and Empowerment fact sheet library topics.**

**General Stroke**

- About Stroke, After Stroke, Stroke Rehabilitation

**Obtaining Good Healthcare Information**

- Communicating with your Loved One’s Healthcare Team, Finding Health Information, My Health*e*Vet

**Understanding How Caring for a Loved One Affects You**

- Changes in Relationships, Caregivers who Work Outside of the Home, Caregiver Stress/Depression, Long-Distance Caregiving

**Caring for Someone with Physical Needs**

- Personal Care, Speech & Communication, Changes in Body Function, Pain, Spasticity, Swallowing, Fatigue, Sleep, Urinary Incontinence, Sex After Stroke

**Caring for Someone with Emotional & Behavioral Needs**

- Coping with Emotional Changes, Depression, Apathy, One-Side Neglect, Cognitive & Memory Problems, Personality Changes, Difficult Behaviors

**Keeping Your Loved One Healthy**

- Managing Medicines, Healthy Eating & Exercise, Spirituality

**Helping Your Loved One Become More Independent**

- Preventing Falls, Ways to Make Home Safer, Assistive Devices, Driving & Transportation

**Finding Community Resources**

- Community Services, Getting Help, Stroke Support Groups, Respite Care, Long-Term Care, End of Life Care

**Managing Financial & Legal Issues**

- Finances, Help with Legal Matters, Paying for Community Services

**Helpful Tools**

- Aphasia Card, Medication Card, Personal Health Record

#### Intervention Sessions

The first intervention component, the orientation session, is conducted in one session over the telephone the week following the baseline assessment. Before participating in this session, the caregiver receives in the mail a workbook that provides information about the study and how to access and navigate the website. The goal of this first session is to develop rapport and to orient the caregivers to the RESCUE website and intervention. The nurse provides a guided tour of the RESCUE website and asks the caregivers for a return demonstration of navigating and finding specific information on the website. Similar to how teachers use Powerpoint (Microsoft Corporation) presentations for classroom instruction, the nurse uses the module and diary on the RESCUE website to teach caregivers the steps of the problem-solving method. With coaching and feedback from the nurse, the caregiver develops their personalized problem-solving plans. Last, the nurse summarizes the training, answers questions, and orients the caregivers to the RESCUE messaging center and the follow-up messaging center sessions. Telephone orientation session goals and activities are presented in Textbox 2.

**Intervention session objectives and activities.**

**Telephone orientation session**

- Rapport building and caregiver assessment
  Discussion of changes in stroke survivor
  Assessment of caregiver skills and needs
- Website orientation
  Workbook review
  Website tour
- Review problem-solving module on the Resources and Education for Stroke Caregivers’ Understanding and Empowerment (RESCUE) website
  Discussion of the basic concepts of the problem-solving approach (Creativity, Optimism, Planning, and Expert Information)
- Apply the problem-solving approach and the RESCUE website to address a common caregiver problem
  Illustrative example on caregiver depression and stress
  Nurse demonstrates application of problem-solving approach
- Development of a personalized problem-solving plan
  Nurse guides caregiver in identifying and prioritizing problems
  Review of fact sheet(s) tailored to the caregiver’s problem
  Caregiver works with nurse to develop problem-solving plan
- Summary of the problem-solving approach and messaging center
  Nurse summarizes the session and answers questions
  Nurse demonstrates how to use the RESCUE messaging center

**Messaging center sessions**

- Assess for changes
  Nurse assesses caregiver and stroke survivor status and identifies any changes since previous session
- Review educational material
  Nurse assesses comprehension of assigned fact sheets
- Review discharge plan
  Nurse asks tailored questions pertaining to stroke survivor’s discharge plan
- Apply problem-solving approach
  Nurse providers feedback on caregiver’s previous worksheet
  Nurse asks tailored follow-up questions on caregiver’s problem-solving plan
- Identify new problems (optional)
  Caregiver identifies a new problem they would like to work on (if applicable)
  Caregiver applies problem-solving approach to the new problem
- Prepare for next session
  Nurse assigns fact sheet for next session

The second component, the messaging center component, consists of 8 asynchronous sessions that are conducted over the RESCUE messaging center. The sessions reinforce, sustain, and supplement what was learned during the orientation session component. The goals of the messaging center component are to refresh the caregiver’s knowledge of the problem-solving method, motivate and empower the caregivers’ abilities to access information on the RESCUE website to resolve their problems, and provide additional skills training to facilitate caregivers’ successful adjustment after the stroke survivors’ return to home.

The messaging center sessions involve three parts: (1) education on the RESCUE website, (2) problem-solving planning, and (3) discharge plan review. For part one, caregivers read assigned fact sheets on the RESCUE website that focus on general caregiving skills needed by all caregivers as well as fact sheets that are directly targeted to the unique needs that are identified by the caregivers. The nurse will respond each week with targeted questions about the assigned fact sheets to ensure the caregiver is receiving and understanding the intended information. The nurse also answers any questions from the caregiver and assesses the health and well-being of the stroke survivor and caregiver. In the second part of each session, the caregivers apply their skills training to develop personalized problem-solving plans using the RESCUE messaging center. The role of the nurse is to empower the caregivers via the messaging center to solve their problems by helping them apply their problem-solving training. The third part of each session covers the stroke survivors’ discharge plan. The nurse provides tailored questions pertaining to progress on discharge goals (ie, follow-up appointments, obtaining durable medical equipment). The messaging center session goals and activities are presented in Textbox 2.

The messaging center is a secure site that is located behind the VA firewall. The messaging center uses structured electronic worksheets with a free-text format that enables the caregivers and the nurse to asynchronously communicate online over an encrypted channel. The messaging center is designed to be simple and user-friendly. The caregiver-nurse communication reinforces information that the caregiver has learned and empowers caregivers to act on their behalf, by providing acceptance and integration of new knowledge and reinforcing positive problem-solving skills. For the messaging center sessions, the study team maintains records of “exact correspondence” of the caregiver-nurse communications on the messaging center. We collect data on the amount of time caregivers report spending on the intervention to monitor participant adherence.

The first messaging center session is arranged during the orientation session. Sessions are due weekly, which is communicated to the caregiver in the orientation session and in each messaging center worksheet. A reminder call is placed if the session is not complete the day before it is due. Up to three reminder calls are placed if the session is overdue. If we are unable to reach the caregiver after three attempts, they are withdrawn from the study. If a caregiver is unable or unwilling to continue with the intervention, they are offered the opportunity to discontinue the intervention but remain in the study for data collection phone calls. If a caregiver falls more than 2 weeks behind, the case is discussed at weekly study team meetings. The principal investigator makes the final determination of whether to continue or discontinue the intervention with the participant. Any deviations from the intervention schedule are documented.

### Usual Care Arm

For caregivers in the usual care group, no changes are made in the information, discharge planning, and care the patients who have had a stroke normally receive. Similar to procedures in the intervention group, the staff document the discharge planning and care received by reviewing the stroke survivors’ electronic records. Caregivers randomized to the usual care group participate in data collections at the same time points as the intervention arm (11 and 19 weeks after baseline). No information about the RESCUE website is provided to the usual care group participants during the study, but it is possible for these participants to access the website, as it is publicly available. To assess for contamination, we ask usual care participants if they have ever visited the RESCUE website during follow-up data collection. After completion of the study, usual care participants are mailed self-help materials along with the RESCUE website URL.

### Fidelity Considerations

The team uses recommendations of Borrelli et al [46] and Burgio et al [47] to lessen problems with fidelity and increase the likelihood that the intervention is delivered consistently. The team uses a pretested, standardized study manual that includes data collection scripts and step-by-step directions for conducting the informed consent procedures and intervention. The team provides extensive training to team members that include role-playing activities and didactic instruction on topics such as motivational interviewing, stress reduction techniques, and caregiving issues.

To monitor the delivery of the intervention, a mental health counselor or mental health nurse practitioner listens to the research nurse’s orientation sessions with their first two assigned caregivers to identify any fidelity issues at the beginning of the project. Following this initial monitoring, we periodically review the remaining sessions. We also ask the research nurses to track the number of minutes they spend with each caregiver and to keep detailed notes of any deviations from the study protocol that occur. We discuss the deliveries of the intervention and evaluate adherence to study protocols, noting all deviations on a form. Feedback is provided to the nurses and additional training is provided if needed.

Similarly, we monitor the messaging center sessions that are conducted via the RESCUE messaging center. The counselor or nurse practitioner reviews the first two “exact correspondences” of the caregiver and nurse to identify initial fidelity issues. After this assessment, the counselor or nurse practitioner periodically reviews the exact correspondences between the caregiver and nurses on the messaging center. The research nurses conducting the messaging center sessions also keep detailed notes of any deviations in the protocol that occur during the messaging center sessions. We review the fidelity check data and provide feedback and training, as needed, to the nurses.

We evaluate “enactment” using an adapted treatment acceptability and enactment tool [48,49]. The tool asks caregivers in the intervention group to rate the amount of information and contact they received during the orientation and messaging center sessions, how much they used the skill-building strategies and the RESCUE website, how helpful the intervention was, and whether their problems were resolved. Each item is scored on a 5-point rating scale. We also ask caregivers in the usual care if they used the RESCUE website and whether their problems were resolved.

### Data Collection

#### Quantitative

Staff members administer study instruments to caregivers via telephone at baseline and then caregivers are randomized to one of the two arms. Two posttests are conducted by blinded staff members at 11 and 19 weeks after baseline assessment. Data collection time points were chosen to assess immediate (ie, 1 week postintervention) outcomes and determine if the effect of the intervention is sustained at 2 months postintervention. The staff also supplement information provided by the caregiver about the stroke survivor (ie, demographics, discharge plans, health care use) by reviewing the stroke survivors’ EHR.

#### Qualitative

Staff members conduct in-depth qualitative interviews with a subsample of 15 caregivers who have completed the intervention. A sample size of 15 was chosen due to the relatively narrow scope of our research question, specificity of our sample, and pilot trial experience [50-52]. To obtain a diversity of caregiver perceptions, we use a maximum variation sampling technique [53,54] to select caregivers with high (≥16) and low (<16) scores on the Center for Epidemiological Studies Depression depressive symptom scale [55]. Interviews are collected via telephone separately from quantitative data. In-depth information from the caregivers’ perspectives of the value, acceptability, and facilitators and barriers of the intervention is obtained. The interviews are digitally recorded and transcribed verbatim.

### Data Management and Quality Control

Trained team members use a standardized manual for data collection. Data collectors record participant responses on paper and then enter them into an online database. Quality checks of the data are collected and entered by staff. Within 2 business days, a study member who did not enter or collect the data checks paper data collection forms against database entries to assure that the information is accurate and no data are missing.

### Blinding

The principal investigator and staff collecting outcome data are blinded in this study. Access to files containing group assignment is restricted to unblinded study team members. Prior to collecting outcome data, study staff remind caregivers that they are not to reveal their group assignment. After collecting outcome data, staff complete a blinding assessment. The blinding assessment will be compared to the actual group allocation to assess the effectiveness of blinding procedures.

### Outcomes

The primary outcome is change in stroke caregivers’ depressive symptoms at 11 and 19 weeks after baseline data collection. Secondary caregiver outcomes are changes in burden, stroke knowledge, positive aspects of caregiving, self-efficacy, perceived stress, health-related quality of life, and satisfaction with care at 11 and 19 weeks after baseline. Secondary stroke survivor outcomes are change in functional abilities at 11 and 19 weeks after baseline data collection and health care use.

#### Measures

We carefully chose measures that have good psychometric properties, are easy to administer, and are relatively short in length to reduce participant burden. An important consideration in our selection was including measures that had been used in our previous caregiver studies so that we could compare our results with existing literature. Two burden measures were used to capture the time and difficulty of specific caregiving tasks [56,57] in addition to caregivers’ general feelings of burden [58]. The measures used are presented in Table 1.

**Table 1:** Outcome measures.

Concept; instrument	Description of instrument	Time (mins)
Stress; Perceived Stress Scale [36]	Changes in perceived stress will be measured by the PSS-4^a^. The 4-item measure assesses stress experienced in the last month on a 5-point Likert scale ranging from 0 (never) to 4 (very often). Scores range from 0-16, with higher scores indicating more stress.	<2
Stroke knowledge; National Institutes of Health Stroke Knowledge Tool [59]	The Stroke Knowledge Tool is adapted from the online quiz developed by the National Institutes of Health. The tool consists of 7-items that ask caregivers about their knowledge of the signs, symptoms, and risk factors of stroke. Items are true/false or multiple choice, with higher scores indicating better stroke knowledge. Scores range from 0 to 7.	<5
Depressive symptoms; CES-D^b^ [55]	CES-D is a 20-item, 4-point Likert scale ranging from never (0) to most of the time (3). Possible scores range from 0 to 60, with higher scores indicating more symptoms. It has been used in numerous studies with caregivers and has good reliability and validity [55,60].	<5
Positive Aspects of Caregiving Scale 11-item [61]	The Positive Aspects of Caregiving Scale is an 11-item, 5-point Likert scale ranging from disagree a lot (1) to agree a lot (5) with a range of 11-55. The scale assesses perceptions of benefits within the caregiving context. The questionnaire has demonstrated good reliability and construct validity [62].	<5
Revised Scale for Caregiver Self-Efficacy [63]	This 15-item tool measures caregivers’ judgments about their ability to perform caregiving tasks. We administer the Obtaining Respite (5 items) and Controlling Upsetting Thoughts About Caregiving (5 items) subscales. Respondents rate their level of confidence for each item from 0 to 100. The scale has shown adequate reliability and construct validity [63].	<5
Caregiver burden – Short Version of the Zarit Burden Interview [58]	Items in this 12-item instrument fall into five categories (health, well-being, finances, social life, and relationship with impaired person). This instrument is scored on a 5-point Likert scale ranging from 0 (never) to 4 (nearly always). Possible scores range from 0 to 48, with higher scores indicating higher burden. The instrument was originally developed to measure dementia caregiver burden but has been used in stroke caregiver studies and is appropriate for other caregiver populations [58,64].	<5
Health-related quality of life; VR-12^c^ [65]	The VR-12 consists of 12-items that measure health-related quality of life. Items are scored on a 3-point or 5-point scale. It consists of physical and emotional scales. Scores for each scale are calculated by using an algorithm. Higher scores indicate better health-related quality of life. This is a widely used tool in stroke caregiver studies and has good psychometric properties [66].	<2
Patient satisfaction; General Satisfaction Subscale of the Patient Satisfaction Questionnaire [67]	The scale consists of 6 items on feelings about the recent medical care they have received. Responses to items range from 1 (strongly agree) to 5 (strongly disagree). Scores range from 6 to 30. The scale has demonstrated excellent reliability and good internal consistency [67].	<5
Oberst Caregiving Burden Scale [56,57]	This instrument uses a 5-point response scale to measure the perceived amount of time spent and the perceived level of difficulty of 15 tasks and activities that caregivers do to help stroke survivors. Each item is scored on a 1-5 scale for difficulty (1=not difficult, 5=extremely difficult) and time (1=no time, 5=a great deal of time). The scale has shown good reliability, construct validity, and content validity [57].	<8
Treatment Acceptability and Enactment Tool (adapted from McLennon et al [48] and Bakas et al [49])	This adapted 9-item tool measures caregivers’ perceptions of the value, helpfulness, and enactment of the intervention using a 5-level Likert scale. The caregivers rate different components of the intervention and indicate how often they visited the Resources and Education for Stroke Caregivers’ Understanding and Empowerment website, how often they used problem-solving strategies, and how many problems they resolved. Higher scores indicate a greater level of acceptability and enactment.	<2
Activity of daily living; Barthel Index [34] (completed by caregiver)	This 10-item tool measures patients’ abilities to perform self-care tasks (eg, feeding, bathing). Response options are scored on 5-point increments (eg, 0=unable, 5=needs help, 10=independent). Total scores range from 0 to 100, with higher scores indicating greater functional abilities. The tool has been reported to have excellent reliability, validity, and adequate responsiveness to change in measuring neurologic physical disability [34,68,69].	<2

^a^PSS-4: Perceived Stress Scale.

^b^CES-D: Center for Epidemiologic Studies Depression.

^c^VR-12: RAND 12-Item Health Survey.

### Data Analysis

#### Caregiver and Stroke Survivor Outcomes

For aims 1-3, the focus of the primary analysis is to examine the effect of the intervention based on “intention to treat.” Data from all the participants will be part of the primary analyses regardless of the actual number of completed messaging center sessions. The effectiveness of the intervention will be examined taking into account real-world compliance factors. As an exploratory study element, the team will assess compliance and attempt to determine its effect on study results. The general linear mixed model for repeated measures will be used to model the follow-up depression and secondary outcome times (11 and 19 weeks after baseline data collection). Of primary interest is the estimated within-site intervention effects at 11 and 19 weeks after baseline data collection, controlling for baseline covariates, and stratifying by site. To control for possible chance sample imbalances resulting from randomization, the model will include covariates for baseline prognostic factors (eg, discharge from hospital, community living center, or rehabilitation facility; caregivers’ relationship to stroke survivor; number of previous strokes), deemed to have significant relationships with the response and groupwise imbalances. Thus, the analyses will be able to compare the groups on the measure of interest while controlling for these factors.

#### Budgetary Impact

We will determine the budgetary impact of the intervention. This analysis will consist of two parts: (1) the incremental cost of the intervention itself over and above usual care and (2) the impact of the intervention on health care use. Microcosting techniques [70] combined with average costing [71] will be used to determine the average staff time, wage, space, and equipment costs associated with the intervention. The microcost estimate for the orientation and messaging center sessions will use the average elapsed time of such sessions along with an estimate of the average national wage of the type of nurse most likely to deliver the intervention in the field. To determine the intervention’s impact on the costs of health care use, the team will rely on the Professional Society for Health Economics and Outcomes Research 2014 budgetary impact analysis guidelines [72]. Data on VA-funded use costs will be obtained from Managerial Cost Accounting System and the Non-VA Medical Care files. The team will tabulate all costs from these sources for study enrollees throughout the study, calculate the difference between intervention and usual care average costs, and test for the statistical significance of this difference using the *z* score method proposed by Zhou et al [73]. The final step in determining the budgetary impact of the intervention will combine parts 1 and 2 to determine the complete impact of the intervention on the VA budget.

#### Qualitative Interviews

Qualitative data will be managed using NVIVO (QSR International). We will use template analysis for qualitative data [74,75]. Template analysis is a method of thematically organizing and analyzing qualitative data. We will develop a coding framework with a priori themes based on the qualitative interview guide. We will apply this framework to the first three interviews and add or revise themes as needed. We will use an iterative process in which team members will independently code each transcript and meet to compare coding and resolve discrepancies. Study team members will continue this process until no new themes are identified. The qualitative team members will interact regularly with the principal investigator and the entire team to discuss the findings and to search for alternative explanations in the data. An audit trail containing a log of all decisions and changes, along with the reason for the decision or change, will be kept to ensure methodological rigor.

### Power Analysis and Rationale for Sample Size

The sample for aims 1-4 consists of 240 caregivers. Assuming a standard deviation of 8.2, 84 subjects per group will achieve 80% power to detect a mean intervention effect size of 3.5 on depression at a 5% significance level. The sample size of 120 per group (ie, intervention group, usual care group) was selected to account for the occurrence of a 30% dropout rate. The dropout rate, expected effect size, and variability were deemed reasonable based on previous literature [49,55,76]. For aim 5, we will select a purposive subsample of 15 caregivers who completed the intervention arm of the study. Typically, 8-12 participants are needed to reach theoretical saturation [53].

### Safety Considerations

The study team includes a mental health counselor who will be involved in all cases where there may be concern about a caregiver’s mental health. In the event that a study team member believes caregivers are experiencing unmanageable stress but not severe stress, the caregiver will be advised to call the stroke survivor’s assigned VA primary care social worker or the primary care clinicians of the patient’s care team. Study staff will also give the caregivers the phone number of the VA Caregiver Support Line and other referral sources as needed. If any study team member feels that a caregiver may be experiencing severe stress or crisis, or if the team member uncovers that the patient who has had a stroke has serious changes in their health, the team member will call a member of the stroke survivor’s VA primary care team or the local emergency response system. In the event that any abuse or neglect is suspected, study personnel will follow established procedures by oversight agencies, including the VA, Institutional Review Board, and the state Abuse Hotline.

## Results

Study enrollment began in June 2015 and is ongoing. As of May 2020, we have enrolled 141 caregivers and 104 caregivers have completed the study. The first results are expected to be submitted for publication in 2021.

## Discussion

### The RESCUE Intervention

It is well documented in the literature that strokes are disabling and require extensive involvement of family caregivers for successful rehabilitation of the stroke survivor [1,3]. Caregivers are often ill-prepared to manage their own problems as well as the multiple psychological, social, and physical disabilities of their stroke survivors. Thus, national organizations emphasize the importance of providing information and support interventions to facilitate the problem-solving skills of caregivers [2,3].

Although tailored support and problem-solving interventions have been effective in improving stroke caregiver outcomes, these interventions are often burdensome to caregivers and not feasible to implement in routine clinical care. The RESCUE intervention was planned to be pragmatic and, if found to be successful, easily implemented in the real-world setting. Use of the asynchronous messaging center minimizes caregiver burden and requires less health care system resources compared to in-person or telephone interventions. This intervention is especially relevant because over 70% of American caregivers use the internet to obtain health information [77]. Although we used a VA platform for this intervention, the RESCUE website is publicly available, and the program could be delivered in other health care systems through patient portals and secure messaging systems.

This study has several strengths. Few stroke caregiving studies have included an aim to assess the budgetary impact of the intervention. Although the study is not powered to conduct a full cost-effectiveness study, our data will provide information on costs that can be used to guide future implementation efforts. The study uses implementation science strategies such as the development of partnerships with VA and community clinicians and leaders to collaborate throughout all the study phases and develop plans for dissemination of findings and strategies to transport the intervention to other sites. The qualitative interviews will additionally provide information for improving and refining the intervention to further increase the likelihood that it will be useful in clinical practice.

### Limitations

Because the study is an RCT, threats to internal validity are less likely to be an issue [78]. However, even with this study design, there are internal threats to validity, such as diffusion of treatment and the Hawthorne effect. External validity threats may jeopardize the generalizability of the study findings. For example, the sample is restricted to caregivers who have a reading level above sixth grade and are capable of navigating the internet. It may be possible that only caregivers who already have problem-solving skills and are confident will choose to participate, thereby further restricting the sample to caregivers who already have self-efficacy and are less in need of the intervention.

Respondent burden is a potential limitation but is minimized by using instruments that are brief and understandable. Data collection sessions are arranged at convenient times and messaging center sessions are conducted asynchronously online whenever the caregivers choose to participate. Except for the budgetary impact, the data are self-reports from caregivers, and thus, there may be errors due to poor recall and caregivers providing socially desirable answers.

A challenge in this study is recruitment and retention. We have experienced lower than expected recruitment that is likely attributable to a variety of factors, including a general decline in admissions for stroke in recent years, VA patients receiving care at non-VA facilities, advancements in stroke care, a lower than expected number of patients who have had a stroke at VA facilities, and potential participants failing to meet eligibility criteria, such as lack of internet access. We sought to address these issues by expanding our recruitment sites; however, recruitment remains a challenge. Other strategies we employed to address recruitment issues include regular clinic visits by the research nurse, presentations about the study to clinicians and other stakeholders, and consultation with the advisory panel. To mitigate retention issues, staff carefully explain how much time and effort is required prior to enrollment and schedule study activities at times convenient to the caregiver. In addition, we mail postcards to enrolled caregivers who the team cannot contact after three attempts. Implementation of the program in routine practice may alleviate some of these issues by removing study-specific eligibility criteria, but the program may also have less reach than originally anticipated.

### Conclusions

This is the first known study to test a transitional care and messaging center educational intervention combined with online training and application of the problem-solving approach to improve the quality of caregiving and the recovery of patients post stroke to enable them to remain at home. Other outcomes will be an updated stroke caregiver website and an evidence-based intervention (transitional care, online training, and messaging between providers and caregivers) that can be transportable to other sites and used as a model to improve caregiving of patients with other chronic diseases.

## Appendix

Multimedia Appendix 1 Peer-review report from HSR-6 Post-acute and Long-term care.

## Abbreviations

ADL: activity of daily living
COPE: Creativity, Optimism, Planning, and Expert Information
EHR: electronic health record
ICD10: International Statistical Classification of Diseases and Related Health Problems, 10th revision
RCT: randomized controlled trial
RESCUE: Resources and Education for Stroke Caregivers’ Understanding and Empowerment
VA: Veterans Affairs
