# Supplementary material for: Postdischarge Intervention for Stroke Caregivers: Protocol for a Randomized Controlled Trial
Source: JMIR Res Protoc. 2020 Nov 11;9(11):e21799. doi: 10.2196/21799 (PMC7688383; doi:10.2196/21799)
Supplement: Multimedia Appendix 1 [file resprot_v9i11e21799_app1.pdf]

SUMMARY STATEMENT

PROGRAM CONTACT: (Privileged Communication) *Release Date:* 04/03/2012

Application Number: 1 I01 HX000834-01A1

Principal Investigator

UPHOLD, CONSTANCE

Applicant Organization: VETERANS HEALTH ADMINISTRATION

Review Group: HSR6
HSR-6 Post-acute and Long-term Care
HSR 6 - Post-acute and Long-term Care

Meeting Date: 03/07/2012 *RFA/PA:* HX12-009
Council: MAY 2012
Requested Start: 07/01/2012

Project Title: Utilizing the RESCUE Stroke Caregiver Website to Enhance Discharge Planning

SRG Action: Priority Score: 169 Percentile: 7.1
Human Subjects: 30-Human subjects involved - Certified, no SRG concerns
Animal Subjects: 10-No live vertebrate animals involved for competing appl.
Gender: 1A-Both genders, scientifically acceptable
Minority: 1A-Minorities and non-minorities, scientifically acceptable
Children: 1A-Both Children and Adults, scientifically acceptable
Clinical Research - not NIH-defined Phase III Trial

Project Year	Direct Costs Requested
1	277,837
2	270,311
3	296,996
4	254,065
TOTAL	1,099,209

ADMINISTRATIVE BUDGET NOTE: The budget shown is the requested budget and has not been adjusted to reflect any recommendations made by reviewers. If an award is planned, the costs will be calculated by VA Office of Research and Development (ORD) staff based on the recommendations outlined in the BUDGET COMMENT section and any relevant ORD service-specific limitations.

NOTE TO APPLICANT: This application is being considered for funding. This is not, however, an indication that a funding decision has been made. You should complete the Just-in-Time process at your local VA Medical Center as soon as possible so that if this proposal is selected for funding there will be no delay in the release of funds. As a reminder, funding is always dependent upon the availability of funds.

KEY SUMMARY POINTS:

1. This is a well-designed study of the proposed intervention, which now includes multiple components that make it potentially sustainable and cost-saving. The applicants have addressed many of the prior concerns related to the intervention intensity.
2. The intervention group will now be compared to an attention control group that includes a messaging component, which includes referral to primary care providers or the VA Caregivers Support Line. Some concerns were expressed about this aspect of the control group, which may lead to a smaller comparative intervention effect. The advantages and disadvantages of different types of control groups -- with or without overlap with the intervention -- should be considered.
3. The research team is experienced and highly qualified, and has done good preliminary work leading to the development of the RESCUE Caregiver website.
4. The use of mixed methods to evaluate the proposed intervention is excellent. There are too few qualitative interviews, however, to reach saturation.
5. The dissemination plan is underdeveloped.

DESCRIPTION (provided by applicant):

Background: Caregiver depression is common following a family member's stroke and is a major contributor of survivor's hospital readmission and institutionalization. Researchers have consistently found that interventions to help caregivers resolve problems are effective in reducing depressive symptoms. However, these problem-solving interventions have been underused in practice because they involve multiple, in-person or telephone sessions and require large amounts of staff time to implement. To overcome these barriers, our long-term goal is to implement stroke caregiver programs that involve low-cost interventions that are sustainable in routine clinical practice. Our immediate objective is to test, using a randomized controlled trial, a problem-solving intervention for stroke caregivers that can be delivered during the Veterans' in-patient stays followed by online, in-home sessions. We will modify the traditional, problem-solving intervention by adding web-based training using interactive modules, factsheets, and tools on our previously developed and nationally available RESCUE Caregiver website (www.rorc.research.va.gov/rescue). We will also provide on-line, skills training and application of the problem-solving approach via the RESCUE messaging center, a secure site behind the VA firewall. This work builds on the team's extensive experience in stroke caregiver education. Our immediate, primary aim (#1) is to test the effect of the intervention on stroke caregivers' depressive symptoms at 14 weeks post-discharge from in-patient settings. Primary Hypothesis: Stroke caregivers who receive the intervention will have less depressive symptoms at 14 weeks compared to stroke caregivers in the attention control group. We propose four secondary aims. Aim #2 is to test the effect of the intervention on stroke caregivers' burden, problem-solving abilities, self-efficacy, health-related quality of life (HRQOL), and satisfaction with care at 14 weeks post-discharge. Aim #3 is to test the effect of the intervention on Veterans' outcomes: functional abilities and healthcare utilization (i.e., unintended hospital bed days of care, number of emergency room visits, number of unscheduled clinic visits) at 14 weeks post-discharge. Aim #4 is to determine the budgetary impact for implementing the intervention. Aim #5 is to determine the facilitators, barriers and best practices for implementing the intervention. Methods: We will conduct a two-group randomized controlled trial with repeated measures and use mixed methods to determine caregivers' perceptions of the intervention. We will enroll 240 stroke caregivers at 3 study sites (North Florida/South Georgia Veterans Healthcare System, Miami VA Healthcare System, and James A. Haley Veterans Hospital in Tampa) in VISN8. Eligible caregivers will be interviewed, complete baseline measures, and then be randomized to two groups: 1) intervention group, or 2) attention control group. A research assistant (RA) will telephone caregivers at 7 weeks and 14 weeks to answer questions on instruments with established reliability and validity. The RA will review the Veterans' VA Computerized Patient Record System health record to obtain information on the Veterans' healthcare utilization. We will determine the budgetary impact of the intervention by

examining the cost data in the Decision Support System National Data Extracts and VA fee-basis files. Qualitative interviews will be conducted with selected caregivers to obtain in-depth perceptions of the value, facilitators, and barriers of the intervention. Throughout all phases of the project, we will collaborate with our VA (Offices of Nursing Service, Office of Geriatrics and Extended Care, My HealtheVet Program Office, Stroke QUERI Center) and non-VA partners (American Stroke Association). Impact: This is the first known study to test a discharge-planning intervention combined with technology to improve the quality of caregiving and the recovery of Veterans. Other outcomes will be a state-of-the-art website and an evidence-based model (in-patient, discharge planning and online, training and caregiver-provider messaging) that can be transportable to other disease models.

CRITIQUE 1

1. Significance of the Specific Aims Proposed.
Criterion Score: 1.2

This multi-site mixed methods study aims to improve both functional and cost outcomes in veterans post-stroke, and stress-related outcomes in their family caregivers, by providing an innovative multi-component adjuvant discharge planning intervention to standard interventions that are usually received by this dyad. It is an innovative technologically based intervention that is responsive to changing caregiver populations and their openness and experience with technology; and is well grounded in Implementation Science.

2. Impact / Innovation / Contribution to VHA.
Criterion Score: 1.2

This study addresses a number of crosscutting HSR&D priority areas: a) healthcare informatics through its inclusion of the RESCUE website and messaging center; b) community-based long-term care and caregiving; and c) family caregiver mental and behavioral health. This multi-component intervention is theoretically based and has a well-defined intervention structure. The success of this program of research is likely to influence the continued development of caregiving science with other chronically ill clinical populations who require the support of family members so care recipients can receive less expensive community-based care, at less cost to their family caregiver's mental and physical health.

3. Approach.
Criterion Score: 1.2

The investigative team has been very responsive to reviewers' prior comments by paying particular attention to the revision and further articulation of the proposed treatment intervention. It uses an eloquent and integrated theoretical approach in further defining the proposed intervention. The study uses a well-reasoned approach in selecting appropriate measures and proposes a clear plan for data analysis and dissemination of findings.

4. Investigator Qualifications / Resources and Environment.
Criterion Score: 1.2

The investigative team has a wide range of experience, and is well connected with participating sites and community-based facilities. The team has implemented and/or pilot-tested all major components of the proposed multi-component intervention. Three additional consultants were added to provide further support for the informatics intervention component.

5. Feasibility.
Investigators have demonstrated the feasibility of implementing the intervention from a sample perspective, intervention resources, through selection of appropriate measures, the data analytic plan and through dissemination of findings into community-based settings.

6. Project Organization and Management.
The project is well organized and the timeline is appropriate for project implementation.

7. Human Subject Protections.
Human subject protections are appropriately articulated.

8. Inclusion of Women and Minorities.
Investigators address numbers of women and multicultural study participants.

9. Response to Previous Review.
The application addresses prior reviewers' concerns, both in the Introduction and throughout the application.

10. Budget.
No further recommendations.

11. Overall Impression.
This is a well-developed study that is likely to be successful and advance the field of caregiving science.

12. Key Strengths.
1. Well-designed study and theoretically based intervention.
2. Highly qualified and experienced research team.
3. High likelihood of advancing caregiving science and implementing study findings.

13. Key Weaknesses.
1. No major weaknesses remain.

CRITIQUE 2

1. Significance of the Specific Aims Proposed.
Criterion Score: 1.75

The proposed research continues to have potential to advance the health and health care of Veterans with stroke and their family caregivers. Improving hospital discharge outcomes of persons with stroke is an important area, and the investigators have now crafted a more intensive, online intervention. As noted in the earlier review, a focus on family caregivers' outcomes during this transition is not as well-studied. The problem-solving approach, coupled with a well-tested web-based platform (at least in terms of its implementation) would yield a potentially significant service for stroke caregivers.

2. Impact / Innovation / Contribution to VHA.
Criterion Score: 2

The project addresses a priority area for HSR&D and/or VHA. As now developed, the proposed project will challenge current research models related to caregiver intervention delivery. There is still some skepticism that the largely education-based format of the web site is less dynamic than it could be. The investigators are to be commended in their improvement of the intervention intensity, however.

3. Approach.
Criterion Score: 2

The overall research plan remains appropriate to the aims of the project. The project is based in a conceptual model of health communication, which has guided the development of the intervention website. The use of mixed methods is well developed and a strength, and now much stronger point of interface is evident (sampling caregivers in the qual component based on their depression scores in the quan component. Several issues remain, however:

1. An outline of the new, more intensive structure of the intervention up front would have led to improved narrative structure of the proposal.
2. A rationale is still required specifying the number of older adults using the internet for health purposes, and how this limits or enhances the feasibility of the proposed intervention (the fairly small-scale implementation studies presented do not).
3. The study flow figure was not readable.
4. It is still not entirely clear how variations in sites will be incorporated into the efficacy analysis.
5. Are there other support programs stroke caregivers could use that could make them ineligible?
6. What if a crisis occurs? Do caregivers have to wait until the next day to receive a message from the research nurse?
7. Instead of having caregivers self-report use, can't the website track this?
8. Again, the measures are not well-aligned with the model.
9. Given the measures included, could clinical cut-points also be considered, such as depression or burden (as both measures have established cut-points)?
10. More detailed data on cost analysis is needed.
11. Could do more than 9 qualitative interviews, as even in a qualitative component this is a small number to reach saturation.
12. Do you mean high and low depression scores at 14 weeks? What about change over time in these measures; couldn't that be used to select participants in the qualitative component?
13. Will caregivers continue to complete follow-up interviews after possible institutionalization?
14. More detail on the dissemination plan is needed.

4. Investigator Qualifications / Resources and Environment.
Criterion Score: 1.4

The research team is appropriate and has a track record for success. The research team has the knowledge/background and resources to ensure timely and successful project completion. The access to three stroke clinic sites is noted. Strong support is also apparent from the applicant's VA facility and collaborators. The PI has strong publication record. The investigators are to be commended for adding new team members, but there is ongoing concern about whether there is enough clinical expertise on the team -- i.e., does anyone on the team actually have expertise providing clinical consultation or care management directly to caregivers, as opposed to delivering educational materials on a website?

5. Feasibility.
The application demonstrates the feasibility of the proposed project, and a detailed table is provided suggesting that the enrollment targets are achievable. The proposed data collection instruments are

reliable and valid. An appropriate quality control procedure is also provided. Although the dissemination plan is limited, the use of CBPR lends confidence to the dissemination of this project if it is implemented.

6. Project Organization and Management.
The large number of Co-Is dedicate very low percent effort.

7. Human Subject Protections.
Appropriate.

8. Inclusion of Women and Minorities.
Appropriate.

9. Response to Previous Review.
The investigators were thorough in addressing the multiple comments of the reviewers.

10. Budget.
The budget is appropriate.

11. Overall Impression.
This is a well-designed study with a strong research team. The use of mixed methods to evaluate the proposed intervention is also excellent. The applicants have addressed many of the major concerns with the prior version (in particular, the intervention intensity), and thus the strengths outweigh the concerns.

12. Key Strengths.
1. Strong research team.
2. Excellent use of mixed methods and community-based participatory approaches to evaluate (and eventually implement) the proposed intervention.
3. Good preliminary work leading the development of the website.

13. Key Weaknesses.
1. Questions are still apparent regarding how often caregivers will actually use the website to address their day-to-day care demands.
2. There are some issues that still remain related to the clinical responsiveness of the system.
3. Some details on project management and dissemination remain lacking.

CRITIQUE 3

1. Significance of the Specific Aims Proposed.
Criterion Score: 1.6

This proposed study uses multi-disciplinary approaches to provide interventions to solve problems to caregivers of stroke patients; the proposed intervention is designed to deliver the intervention at patient's discharge in one session followed by web-based training using interactive modules and problem-solving by online messaging center. If the intervention is shown to be effective, it will provide a potentially cost-saving and sustainable post-discharge strategy for caregivers that would have positive impact on both patients and caregivers.

2. Impact / Innovation / Contribution to VHA.
Criterion Score: 1.7

The proposed intervention has multiple components, including an in-person discharge planning with a problem solving training session, a web-based training modified from the investigators' previously developed RESCUE Caregiver website, and regular use of message center to solve problems. The ultimate goal is to overcome the already identified barrier of large staff time needed to address caregiver problem solving. It is innovative to use the message center to help with caregiver problem solving, and this post-discharge in-home component makes the intervention more individualized, intensive and sustainable. Although it remains to be seen whether the message center will be utilized as regularly, if the message center is utilized as intended, the expectation is that it will save staff time over the more in-person or telephone sessions that may need to be offered during high anxiety periods for caregivers.

3. Approach.
Criterion Score: 1.9

The approaches are carefully and clearly presented.

The revised application added the message center as an in-home component of the intervention and also used an attention control group rather than a usual care control group. The proposal indicates that the attention control group allows the researcher to determine whether improvements in the dependent variable are due to the treatment, or simply to the added attention the participant receives. The idea is to be able to determine if outcomes improve with nurse-caregiver communication alone (attention control group) or if the structured training and problem-solving approach (intervention group) is essential. However, since the attention control group caregivers will not only exchange communications regarding the Veteran's status with a nurse, but will also be referred by the nurse to the primary care providers or the VA Caregiver Support Line, the difference in caregiver anxiety and depression between the two groups may be quite small, especially if the caregivers contact providers or the support center. One wonders if the attention control group was a good choice as a control group since the intervention to be tested is really a multi-component intervention including the use of messaging center. If, on the other hand, attention control group is to be used as a comparator, as indicated above, it is difficult to see caregiver or patient outcome to be as meaningfully larger in the intervention group compared with attention control group, and if the study should now be a non-inferiority trial or an equivalence trial as far as caregiver or patient outcomes are concerned.

4. Investigator Qualifications / Resources and Environment.
Criterion Score: 1.5

The investigators are highly qualified, with extensive experience in caregiver research. The team has also put much effort in partnering and collaborating with various national and local stakeholders.

5. Feasibility.
The application shows the feasibility in getting the needed subjects and adequate data very adequately.

6. Project Organization and Management.
Appropriate and well organized.

7. Human Subject Protections.
Appropriate.

8. Inclusion of Women and Minorities.
Appropriate.

9. Response to Previous Review.
Responses are thorough and well thought out. In response to previous review, the investigators have changed the intervention to have more individualized and interactive in-home components based on discussions with partners and clinicians at study sites. The new design now includes those who are more at risk of developing depression symptoms. They now have a detailed qualitative component.

Minor issue is that the responses to key summary points describe the added details to illustrate how the conceptual models have an impact on outcomes and guide the statistical analysis, but the analysis plan did not include any such details described in the introduction.

Instead of usual care, attention control group will now be the comparative group, and usual care components will be documented throughout the study. This change did not appear to be in response to previous review, and as stated above, it is unclear if the change in control group was a good choice.

10. Budget.
Appropriate.

11. Overall Impression.
The proposal is clearly written, and the investigative team has extensive experience with various components of the proposed intervention and has various pilot studies.

12. Key Strengths.
1. A very well planned study using multi-disciplinary approach.
2. The proposed intervention has multiple components that make it potentially sustainable and cost-saving.
3. Excellent collaboration with partners and stakeholders.

13. Key Weaknesses.
1. It is unclear whether the intervention will be more effective with respect to caregiver and patient outcomes compared with the currently proposed attention control group.

MEETING ROSTER

HSR-6 Post-acute and Long-term Care
Health Services Research Parent IRG
Office of Research & Development
HSR 6 - Post-acute and Long-term Care
HSR6 R
March 07, 2012

CHAIRPERSON

BERLOWITZ, DAN R., MD
DIRECTOR
CTR FOR HLTH QUALITY OUTCOMES & ECONOMIC RES
BEDFORD VA MEDICAL CENTER
PROFESSOR, SCHOOLS OF PUBLIC HLTH AND MEDICINE
BOSTON UNIVERSITY
BEDFORD, MA 01730

MEMBERS

ALESSI, CATHY A., MD
ASSOCIATE DIRECTOR
CLINICAL PROGRAM AND CLINICAL RESEARCH
GRECC, VA GREATER LOS ANGELES HEALTHCARE SYS
PROFESSOR OF MEDICINE
UNIVERSITY OF CALIFORNIA LOS ANGELES
SEPULVEDA, CA 91343

BEATTIE, PAUL F., PHD, PT
CLINICAL ASSOCIATE PROFESSOR
PROGRAM IN PHYSICAL THERAPY
DEPARTMENT OF EXERCISE SCIENCE
UNIVERSITY OF SOUTH CAROLINA
COLUMBIA, SC 29206

BOSWORTH, HAYDEN B., PHD *
ASSOCIATE DIRECTOR
CTR FOR HEALTH SERVICES RESEARCH IN
PRIMARY CARE, DURHAM VAMC
RESEARCH PROFESSOR
DUKE UNIVERSITY SCHOOL OF MEDICINE
DURHAM, NC 27705

BRADHAM, DOUGLAS D., DRPH
HEALTH SERVICES RESEARCH DIRECTOR
WICHITA VA MEDICAL CENTER
PROFESSOR OF PUBLIC HEALTH
DEPT OF PREVENTIVE MEDICINE AND PUBLIC HEALTH
UNIVERSITY OF KANSAS SCHOOL OF MEDICINE
WICHITA, KS 67214

CHODOSH, JOSHUA , MD *
ASSISTANT CHIEF OF STAFF
PATIENT SAFETY / QUALITY MANAGEMENT
VA GREATER LOS ANGELES HEALTH CARE SYSTEM
ASSOCIATE PROFESSOR OF MEDICINE
UNIVERSITY OF CALIFORNIA LOS ANGELES
LOS ANGELES, CA 90073

DUBBERT, PATRICIA M., PHD, BSN
ASSOCIATE DIRECTOR FOR IMPROVING CLINICAL CARE
MENTAL ILLNESS RESEARCH, EDUCATION
AND CLINICAL CENTER
LITTLE ROCK VA MEDICAL CENTER
LITTLE ROCK, AR 72205

FARRAN, CAROL J., RN, DNSC *
PROFESSOR
ADULT HEALTH AND GERONTOLOGICAL NURSING
COLLEGE OF NURSING, GRADUATE COLLEGE
RUSH UNIVERSITY MEDICAL CENTER
CHICAGO, IL 60612

FINDLEY, THOMAS W., MD, PHD
RESEARCH CHAIR
INTEGRATIVE MEDICINE COMMITTEE
EAST ORANGE VA MEDICAL CENTER
PROFESSOR, DEPT OF PHYSICAL MEDICINE AND REHAB
UMDNJ - NEW JERSEY MEDICAL SCHOOL
EAST ORANGE, NJ 07018

GAUGLER, JOSEPH E., PHD
ASSOCIATE PROFESSOR
SCHOOL OF NURSING, CENTER ON AGING
UNIVERSITY OF MINNESOTA
MINNEAPOLIS, MN 55455

HUTT, EVELYN A., MD
PROGRAM DIRECTOR
COLORADO REAP TO IMPROVE CARE
COORDINATION
ASSOCIATE PROFESSOR
UNIVERSITY OF COLORADO SCHOOL OF MEDICINE
DENVER, CO 80220

JONES, KATHERINE R., RN, PHD *
PROFESSOR AND ASSOCIATE DEAN
FRANCES PAYNE BOLTON SCHOOL OF NURSING
CASE WESTERN RESERVE UNIVERSITY
CLEVELAND, OH 44106

KIM, HYUNGJIN MYRA, PHD
ASSOCIATE RESEARCH SCIENTIST
CTR FOR STATISTICAL CONSULTATION AND RESEARCH
ADJUNCT ASSOCIATE PROFESSOR
DEPARTMENT OF BIOSTATISTICS
UNIVERSITY OF MICHIGAN
ANN ARBOR, MI 48109

MATHENY, MICHAEL E., MPH, MD *
STAFF PHYSICIAN AND ASSOCIATE DIRECTOR
BIOMEDICAL INFORMATICS FELLOWSHIP
VA TENNESSEE VALLEY HEALTHCARE SYSTEM
ASSISTANT PROFESSOR OF MEDICINE
VANDERBILT UNIVERSITY
NASHVILLE, TN 37240

PENROD, JOAN D., PHD
RESEARCH HEALTH SCIENTIST AND DIRECTOR
VA CTR FOR STUDY OF HEALTH CARE ACROSS SYSTEMS
AND SITES OF CARE, BRONX VA MEDICAL CENTER
ASSOCIATE PROFESSOR, GERIATRICS & PALLIATIVE MED
MOUNT SINAI SCHOOL OF MEDICINE
BRONX, NY 10468

SNOW, ANDREA LYNN, PHD
CLINICAL RESEARCH PSYCHOLOGIST
TUSCALOOSA VAMC
ASSOCIATE PROFESSOR, CENTER FOR MENTAL HEALTH
& AGING, AND THE DEPT OF PSYCHOLOGY
UNIVERSITY OF ALABAMA
TUSCALOOSA, AL 35422

SULLIVAN, DENNIS H., MD
DIRECTOR
GERIATRIC RESEARCH EDUCATION AND CLINICAL CTR
CENTRAL ARKANSAS VETERANS HEALTHCARE SYSTEM
PROFESSOR OF GERIATRICS AND INTERNAL MEDICINE
UNIVERSITY OF ARKANSAS FOR MEDICAL SCIENCES
LITTLE ROCK, AR 72205

MAIL REVIEWER(S)

RITCHIE, CHRISTINE S., MD
PROFESSOR
CLINICAL TRANSLATIONAL RESEARCH IN AGING
DEPT OF MEDICINE, DIV OF GERIATRICS
UNIVERSITY OF CALIFORNIA, SAN FRANCISCO
SAN FRANCISCO, CA 94118

SCIENTIFIC REVIEW ADMINISTRATOR

HOLDEN, JOHN P., PHD
SCIENTIFIC PROGRAM MANAGER
OFFICE OF RESEARCH AND DEVELOPMENT
VETERANS HEALTH ADMINISTRATION
DEPARTMENT OF VETERANS AFFAIRS
WASHINGTON, DC 20420

* Temporary Member. For grant applications, temporary members may participate in the entire meeting or may review only selected applications as needed.

Consultants are required to absent themselves from the room during the review of any application if their presence would constitute or appear to constitute a conflict of interest.
